# Resistive and New Optical Switching Memory Characteristics Using Thermally Grown Ge_0.2_Se_0.8_ Film in Cu/GeSe_x_/W Structure

**DOI:** 10.1186/s11671-015-1090-1

**Published:** 2015-10-07

**Authors:** Debanjan Jana, Somsubhra Chakrabarti, Sheikh Ziaur Rahaman, Siddheswar Maikap

**Affiliations:** Thin Film Nano Technology Laboratory, Department of Electronic Engineering, Chang Gung University, 259 Wen-Hwa 1st Rd., Kwei-Shan, Tao-Yuan, 333 Taiwan

**Keywords:** Optical switching, Resistive switching, Light illumination, CBRAM, GeSe_x_

## Abstract

It is known that conductive-bridge resistive-random-access-memory (CBRAM) device is very important for future high-density nonvolatile memory as well as logic application. Even though the CBRAM devices using different materials, structures, and switching performance have been reported in Nanoscale Res. Lett., 2015, however, optical switching characteristics by using thermally grown Ge_0.2_Se_0.8_ film in Cu/GeSe_x_/W structure are reported for the first time in this study. The Cu/GeSe_x_/W memory devices have low current compliances (CCs) ranging from 1 nA to 500 μA with low voltage of ±1.2 V, high resistance ratio of approximately 10^3^, stable endurance of >200 cycles, and good data retention of >7 × 10^3^ s at 85 °C. Multi-steps of RESET phenomena and evolution of Cu filaments’ shape under CCs ranging from 1 nA to 500 μA have been discussed. Under external white-light illumination with an intensity of 2.68 mW/cm^2^ (wavelength ranges from 390 to 700 nm), memory device shows optical switching with long read pulse endurance of >10^5^ cycles. This CBRAM device has optically programmed and electrically erased, which can open up a new area of research field for future application.

## Background

Recently, the conductive-bridge resistive-random-access-memory (CBRAM) device is considered among the most promising solutions for future low-cost embedded non-volatile memories [1–5]. Although several solid-electrolyte materials such as GeS_2_ [6], GeTe [7], Ag_2_S [8, 9], and GeSe [10–13] have been reported to explore CBRAM performances and switching mechanism, however, light-induced resistive switching phenomena of different materials have been reported few. Sun et al. [14] have reported white-light illuminated resistive switching behavior using Ag/NiWO_4_/Ti structure. The device is operated under a current of 50 μA. Mou et al. [15] have investigated light illumination effect on Ag/Ag_2_S/Au CBRAM device. It has been reported that turn-off voltage decreases from −0.8 to −0.25 V which might be effect of change of reduction potential of Ag ion under external light. In addition, Retamal et al. [16] have reported the unipolar resistive switching characteristics and variation reduced of resistance states of Pt/ZnO/Pt structure under ultraviolet light illumination with a high RESET current of 5 mA. Liu et al. [17] have reported unipolar resistive switching characteristics using ITO/HfO_x_/TiN structure under ultraviolet light exposure with a high RESET current of >5 mA. The resistive switching phenomena occur owing to oxygen vacancy generation during light illumination. It is realized that a study on resistive switching phenomena of solid-electrolyte material under external light is very important to design high-density memory in future. According to this, we have also reported impact of white-light illumination on GeSe_x_-based CBRAM devices previously [18]. However, resistive switching phenomena and new optical switching by using thermally grown Ge_0.2_Se_0.8_ material in Cu/GeSe_x_/W structure have been reported here. The memory device shows bipolar resistive switching phenomena with CCs ranging from 1 nA to 500 μA under small operation voltage of ±1.2 V, high resistance ratio of approximately 10^3^, good endurance of >200 cycles, and good data retention of >7 × 10^3^ s at 85 °C. Multi-step RESET characteristics and filaments’ shape with CCs ranging from 1 nA to 500 μA have been explained. The device structure and GeSe_x_ film are confirmed by transmission electron microscope (TEM) and energy-dispersive X-ray spectroscopy (EDX) analysis. After white-light illumination with an intensity of 2.68 mW/cm^2^ on the Cu electrode of the via-hole region, optical switching is observed owing to Cu ion migration through GeSe_x_ solid electrolyte as well as stronger Cu filament being formed. Memory device performs good data retention and long read pulse endurance of >10^5^ cycles after white-light illumination.

## Methods

**Fig. 1:**
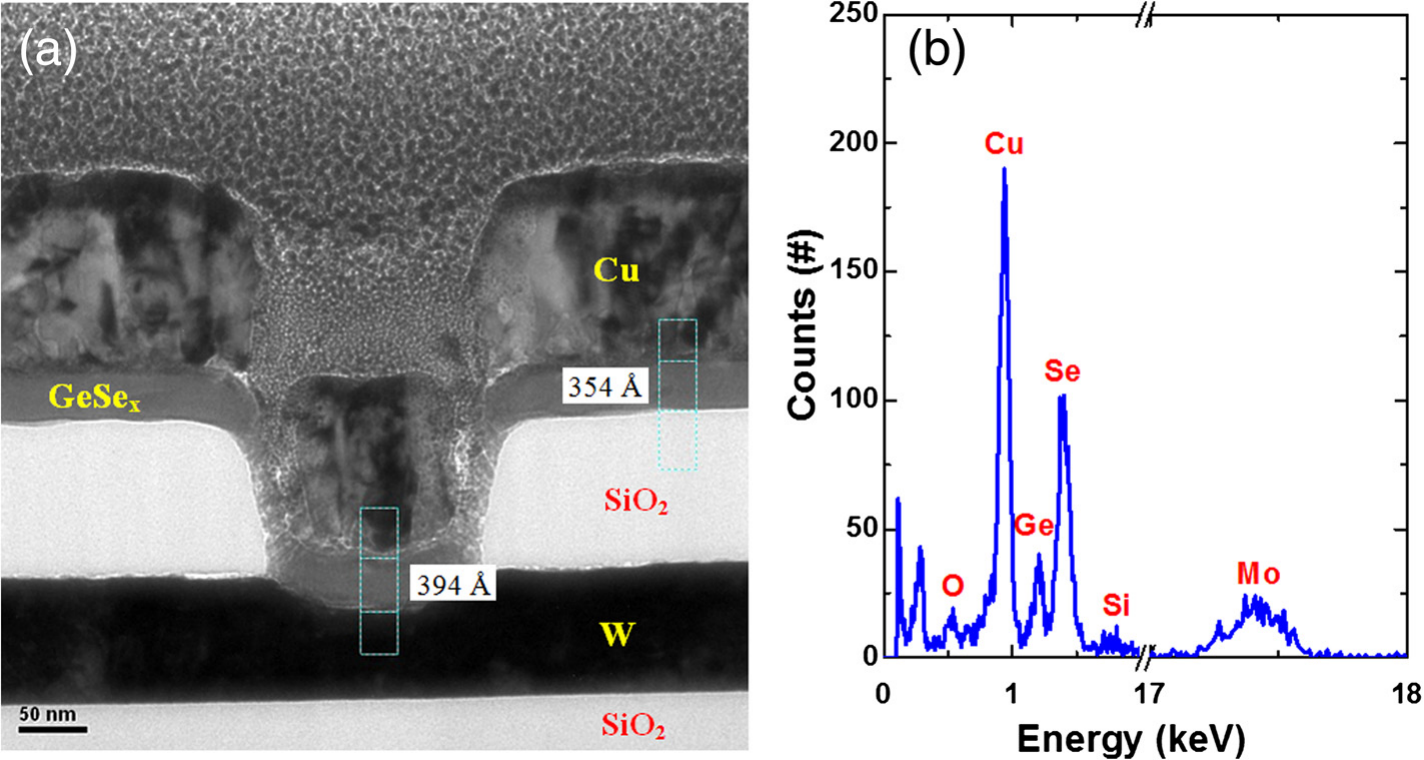
TEM and EDX analysis. **a** TEM image of Cu/GeSe_x_/W CBRAM device. **b** Energy-dispersive X-ray spectrum shows presence of Ge and Se elements in GeSe_x_ film

Tungsten (W) with a thickness of 100 nm was deposited on a SiO_2_/Si wafer. To form via-hole devices, a SiO_2_ layer with a thickness of 150 nm was deposited on W bottom electrode (BE). The via-holes were fabricated by using standard lithography and etching processes. To follow the lift-off process, the photoresist was coated after formation of the via-holes. Then, the Ge_0.4_Se_0.6_ solid-electrolyte material with a thickness of approximately 40 nm was deposited by thermal evaporator. Prior to deposition of GeSe_x_ film, the chamber pressure was 5 × 10^−6^ Torr. The small pieces of Ge_0.4_Se_0.6_ were used for evaporation. It is realized that the percentage of selenium was greater than 60 % in the GeSe_x_ solid electrolyte (or Se-rich GeSe film) because melting point of Se is much lower than the Ge (221 °C vs. 938.2 °C). A copper (Cu) film with a thickness of approximately 150 nm was deposited in situ by the same thermal evaporator. Finally, lift-off process was performed to fabricate the Cu/GeSe_x_/W device. Figure 1a shows a TEM image of Cu/GeSe_x_/W CBRAM device. To confirm the deposition of GeSe_x_ film, a typical size of 0.2 × 0.2 μm^2^ is shown. The layer-by-layer structure is observed clearly. The thicknesses of GeSe_x_ and Cu layers are found to be approximately 39 and 150 nm, respectively. The thickness of W electrode is approximately 90 nm. Figure 1b shows EDX spectrum of GeSe_x_ layer which confirms the Ge and Se contents. Memory characteristics were measured by using Agilent 4156C semiconductor parameter analyzer. White light through optical microscope was applied vertically on Cu TE of the via-hole region. The light intensity was 2.68 *mW/cm*^*2*^ and visible wavelengths were *390*–700 nm. When the device is under illumination, the main portion of GeSe_x_ film which was covered by Cu is under darkness because the light was illuminated on Cu TE. However, the light could be transmitted through thinner sidewall of via-hole to the GeSe_x_ film. After applying light on HRS of the CBRAM devices, the RESET current was measured by applying negative sweeping bias on the Cu TE. The bias was applied on the Cu TE, and W BE was grounded during measurement.

## Results and Discussion

**Fig. 2:**
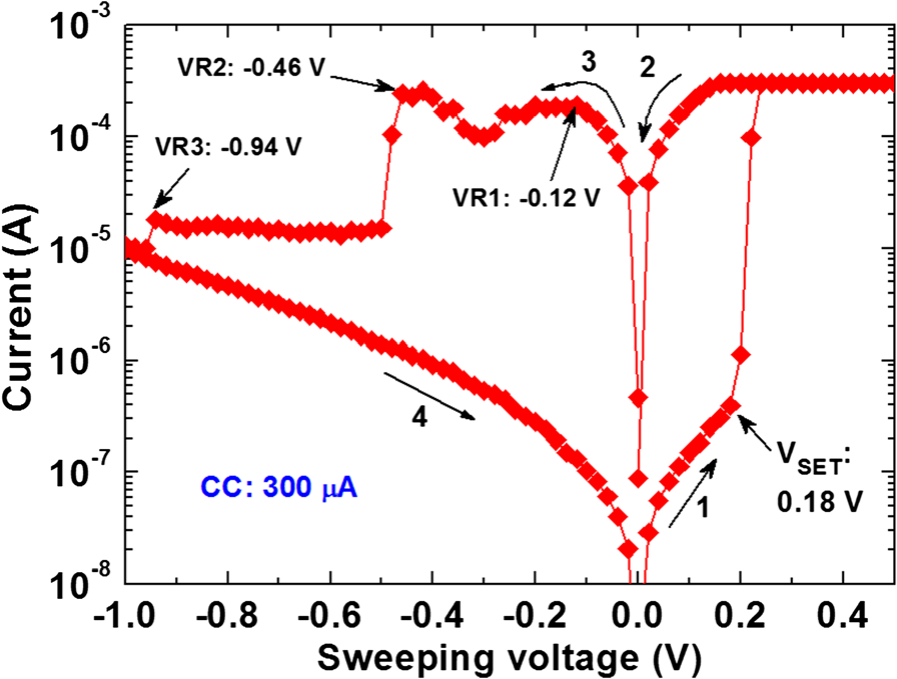
Current–voltage characteristics. Current–voltage hysteresis phenomena of thermally deposited GeSe_x_ film in Cu/GeSe_x_/W structure. Multi-step RESET characteristics are observed owing to conical-shaped Cu filament is formed after CC of 300 μA

Figure 2 shows typical current–voltage (*I-V*) characteristic of the Cu/GeSe_x_/W memory device with a device size of 2 × 2 μm^2^. The sweeping voltage is 0 V → +1 V → 0 V → −1 V → 0 V, which is shown by arrows 1 → 4. This device is operated with a small voltage of ±1.2 V and the current compliance (CC) is 300 μA. For this device, forming process is not needed because GeSe_x_ is solid electrolyte and the film contains a porous material. Therefore, Cu ions can be migrated easily under external low bias as well as formation process can be exempted. A low RESET current (*I*_RESET_) of approximately 190 μA is observed. Both the hold and delay times were 100 μs. The SET voltage (*V*_SET_) is 0.18 V, which is almost the same value for a Cu/Ge_0.2_Se_0.8_/W structure [19]. The values of HRS and LRS are found to be 7.2 MΩ and 0.51 kΩ at a read voltage of 50 mV, respectively. A high resistance ratio (HRS/LRS) of >10^3^ is obtained, which is very useful for high-density memory application. By applying a small *V*_SET_ of >0.18 V on the Cu electrode, the Cu ions are generated at the GeSe_x_/Cu interface and migrated towards the W BE under electric field. The Cu metal starts to grow from the W BE to form conical-shaped metallic Cu filament in the GeSe_x_ solid electrolyte. As a CC of 300 μA, the conducting filament has a shape of conical or pyramid type. The base of filament is on the inert electrode surface and the neck is at the GeSe_x_/Cu interface. By applying negative bias on Cu electrode, this device shows multiple RESETs at VR1 = −0.12 V, VR2 = −0.46 V, and VR3 = −0.94 V. At −0.12 V, an electrochemical oxidation (Cu → Cu^z+^ + ze^−^, where *z* = 1 or 2) process has been started at the GeSe_x_/Cu interface through joule heating, which results in Cu filament starting to dissolve. The current is also decreasing up to −0.3 V because Cu ions are migrated towards Cu electrode as well as the dissolution length of filament is increased. As long as there is sufficient gap in between Cu and filament at a voltage of −0.3 V, more Cu ions will be also generated from the GeSe_x_/filament interface because the diameter is increased towards the W electrode by applying higher negative bias of <0.3 V. In this case, huge Cu ions can be gathered at the Cu/GeSe_x_ interface, which results in a re-growth of filament as well as current increasing up to VR2 of −0.46 V. The re-growth phenomena of a metallic filament are also reported in our previous study by using GeO_x_ or Ta_2_O_5_ material [20]. By applying higher negative voltage of <−0.46 V, a strong electrochemical oxidation with joule heating happened and LRS changes to HRS. The dissolved length of conducting filament is gradually increased up to VR3 of −1 V. Still, the filament remains because of the conical shape. *I-V* curve shows that LRS is ohmic behavior (IαV) whereas HRS follows trap-assisted space charge-limited current conduction behavior (IαV and IαV^2^). It is realized that the shape of Cu filament changes with different CCs, which is also important to understand and this has been explained below.

**Fig. 3:**
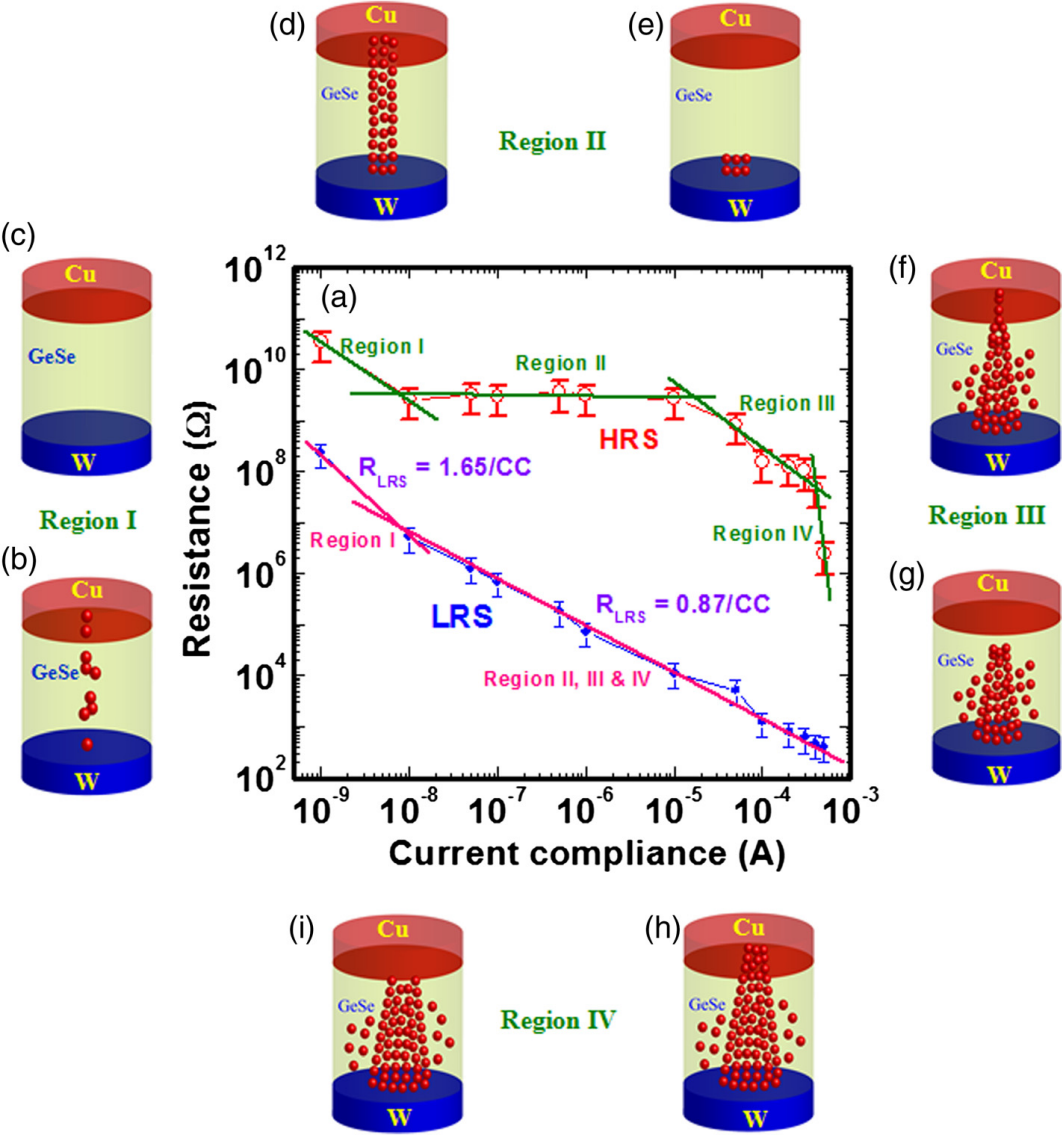
HRS/LRS vs. current compliance. **a** Distribution of HRS and LRS with different current compliances ranging from 1 nA to 500 μA is shown. Different shapes of the Cu filaments are identified by observing the current compliance dependent of both LRS and HRS. Evolution of Cu filament formation and dissolution with different CCs is shown schematically in (**b–i**). At region I, a Cu nano-chain is formed under **b** SET and **c** dissolution under RESET. At region II, a cylindrical filament could be **d** formed and **e** under SET and RESET, respectively. At region III, a conical filament could be formed in the GeSe_x_ film and dissolved at the Cu/GeSe_x_ interface under **f** SET and **g** RESET conditions. **h** A stronger conical-shaped filament could be expected at higher CC of 500 μA under SET. **i** This stronger filament will be dissolved at the Cu/GeSe_x_ interface under RESET

**Fig. 4:**
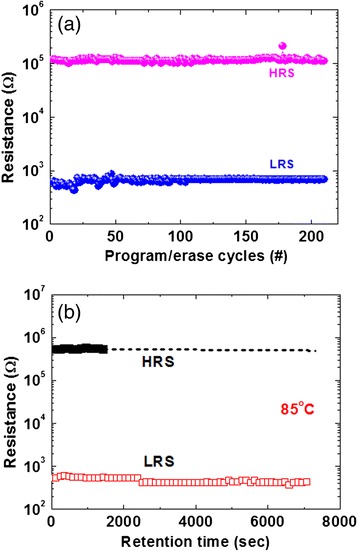
Endurance and data retention characteristics**. a** Stable P/E endurance of >200 cycles of the Cu/GeSe_x_/W structure is obtained. **b** Good data retention characteristics of >7000 s at 85 °C are observed

Figure 3 represents the CC dependence HRS and LRS distribution. The current is varied from 1 nA to 500 μA. Schematic views of different-shaped filaments are also shown. Both values of LRS and HRS are decreased with increasing CC owing to stronger and different-shaped metallic filaments (Fig. 3a). Two distinct regions of LRS are observed. Region I is defined with CCs from 1 to 10 nA (*R*_LRS_ = 1.65/CC). In region II, the current is varied from 10 nA to 500 μA (*R*_LRS_ = 0.87/CC). In the case of HRS, current compliance depends on four regions where “region I” is defined from 1 to 10 nA, “region II” from 50 nA to 10 μA, “region III” from 50 to 300 μA, and “region IV” from 400 to 500 μA. It is assumed that different shapes of filament are formed after SET, which will create asymmetric HRS distribution after RESET operation. Therefore, the filament shapes are created artistically by relative observation of HRS values. In region I, the values of both HRS and LRS are quite high which may cause that continuous Cu metallic filament will not be formed at a low current of <10 nA. A chain-type filament is possible to form or Cu nanocrystals can be formed in the GeSe_x_ solid electrolyte under SET (Fig. 3b). A silver (Ag) nanocrystal filament in SiO_x_ or AlO_x_ film was also reported by Yang et al. [21]. The value of LRS is higher owing to filament resistance added with bulk resistance of GeSe_x_ solid electrolyte. At CC of 1 nA, total filament is dissolved after RESET because the value of HRS is almost the same with pristine one (Fig. 3c). However, the values of both HRS and LRS decrease up to CC of 10 nA and a small filament length remains after RESET. In region II, the value of LRS decreases whereas HRS is independent of CCs. This implies that a cylindrical filament is formed up to CC of 10 μA, as shown in Fig. 3d. Only a small length of filament remains after RESET, as shown in Fig. 3e. In region III, the value of HRS decreases again up to CC of 300 μA owing to conical-shaped filament (Fig. 3f), as mentioned in *I-V* characteristics above. After RESET, the neck side of this conical-shaped filament is dissolved, i.e., a longer length of filament remains on the W BE (Fig. 3g). That is why the value of HRS is decreased. This conical-shaped Cu filament is observed in AlO_x_-based material by Celano et al. [22]. This is like an interface-type switching because the filament is formed/dissolved at the GeSe_x_/Cu interface. In region IV, the value of LRS is decreased by increasing CC up to 500 μA because the conical-shaped diameter is increased further (Fig. 3h). However, the HRS is decreased too after RESET (Fig. 3i). This suggests that the leakage current through the dissolved region is increased because of larger diameter of the remaining filament. In this case, resistive ratio will be controlled by forming/dissolving the metallic filament at the GeSe_x_/Cu interface. A higher resistance ratio of approximately 10^3^ can be observed from CC of 10–300 μA. This CBRAM device has stable program/erase (P/E) endurance of >200 cycles and good data retention of >7000 s at 85 °C, as shown in Fig. 4. Low P/E voltages and currents were +1/−1.2 V and 500 μA/1 mA, respectively (Fig. 4a). A read voltage is 50 mV. The P/E pulse width is 500 μs. Stable resistance ratio of >200 is obtained, which is lower than the mentioned as in *I-V* characteristics. Generally, the value of HRS is decreased after few cycles, as reported previously [12], however this thermally grown GeSe_x_ film has benefit to have unchanged HRS even after 200 cycles. To obtain stable program/erase (P/E) cycles, good structure with a switching material is necessary to design, which is observed here. By adjusting the P/E operation conditions, the filament length or maintaining dissolution gap is also important where the resistance ratio could be decreased because HRS value will be lower. Stable P/E cycles are obtained because major contribution is switching material in a designed structure. Therefore, the stable HRS characteristic is observed because of this thermally grown GeSe_x_ film in the Cu/GeSe_x_/W structure. It is also possible to have different stoichiometry GeSe_x_ film deposited by thermal evaporation which has also key role to have stable HRS. The retention characteristics of our resistive switching memory device are shown in Fig. 4b. An unchanged resistance ratio of approximately 10^3^ at 85 °C has been observed after 7000 s owing to the strong Cu metallic filament formation into the GeSe_x_ solid electrolyte. It is true that HRS value is stable after 1500 s. Due to time limitation of the HP 4156C system, longer time is not dedicated for evaluation. It is interesting to note that this CBRAM device can be programmed by using external white light and erased by using negative bias on the Cu electrode, which have been discussed for the first time below.

**Fig. 5:**
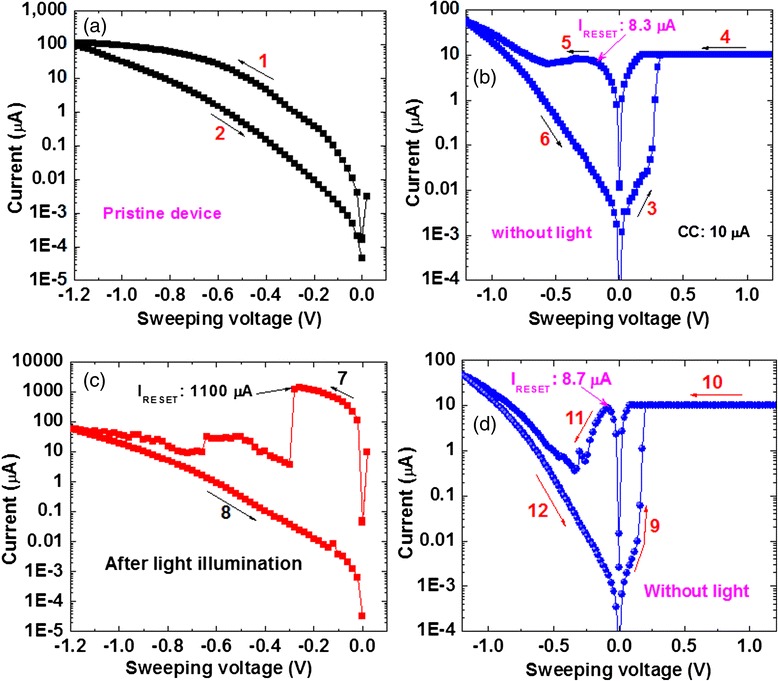
White-light-induced resistive switching memory characteristics. **a** *I-V* characteristics of a pristine device under negative bias. **b** *I-V* hysteresis with a small CC of 10 μA without external light. **c** RESET current characteristics are shown after white-light illumination. **d** *I-V* hysteresis characteristics are shown similar of (**b**) after light turned off condition

**Fig. 6:**
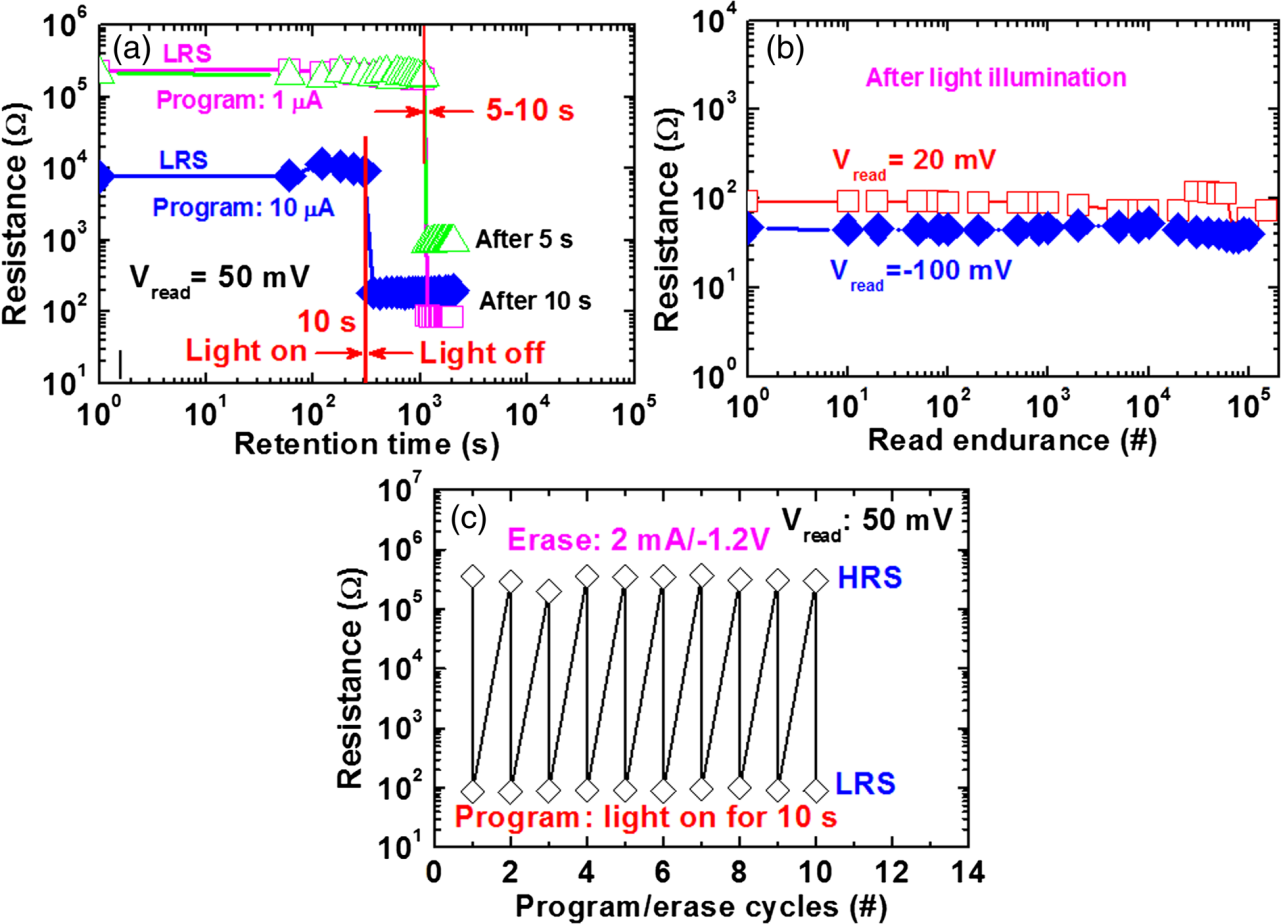
Data retention and read endurance after light illumination. **a** Both values of LRS after programming at CC of 1 and 10 μA are decreased when external white light is turned on with time duration of 5–10 s, which is owing to stronger Cu filament being formed. **b** Read pulse endurance of >10^5^ cycles is observed after light illumination. **c** The device shows program/erase endurance by external light illumination of 10 s and negative erase voltage

Figure 5 shows *I-V* characteristics with and without external white light on Cu/GeSe_x_/W CBRAM device. Evolution of *I-V* hysteresis measurement is shown by arrows from 1 → 12. Figure 5a shows the initial *I-V* characteristics at negative region without light or pristine state. Even though there is no prominent RESET current, however, leakage current is decreased after applying negative bias of −1.2 V (i.e., arrows 1 → 2). This suggests that there is some diffusion of Cu without bias of >*V*_SET_ or external light. It indicates that the device is keeping in environment some days and Cu diffusion may be possible. Figure 5b shows *I-V* characteristics with a low CC of 10 μA and low operation voltage of ±1.2 V is applied with sweeping direction 3 → 6 (i.e., 0 V → +1.2 V → 0 V → −1.2 V → 0 V). A low RESET voltage of −0.18 V and *I*_RESET_ of 8.3 μA are observed. Now, this device is at HRS and white light is turned on for approximately 10 s. Then, it is turned off. All measurements were performed at room temperature (25 °C). *I-V* hysteresis characteristics are shown by arrows 7 → 8 (i.e., 0 V → −1.2 V → 0 V), as shown in Fig. 5c. The RESET voltage (VR1) and *I*_RESET_ are found to be −0.3 V and 1100 μA, respectively. It implies that external light can influence the Cu diffusion into GeSe_x_ solid electrolyte and makes stronger Cu metallic filament from BE to TE resulting to memory device switches from HRS to LRS. Light stimulates the Cu/GeSe_x_ interface and Cu ions are created. The Cu ions have been diffused by this stimulated light energy. Therefore, this needs longer time of >5 s from higher resistance value to lower resistance value. Due to our limitation of lab facility, one light source with a high intensity of 2.68 mW/cm^2^ was used. Therefore, more Cu diffusion into the GeSe_x_ film as well as stronger filament is formed. Further study is needed to control Cu diffusion as well as filament diameter or RESET current could be controlled. Obviously, if the LRS value is low, then the RESET voltage will be also small; especially RESET voltage for the CBRAM devices is small [1]. A large resistance ratio of >10^5^ is observed because of higher RESET current. Due to this high resistance ratio, multi-level cell operation can be obtained by controlling external light. Again, the device shows also normal bipolar resistive switching characteristics without light, as shown in Fig. 5d. This indicates that this device can be programmed by external white light and erased by external negative bias on the Cu TE. Therefore, optical switching is observed and it may open up new research field for external light effect on the CBRAM devices. This optical switching is nonvolatile. The retention characteristics of the Cu/GeSe_x_/W CBRAM devices are shown in Fig. 6a. Stable retention characteristics are observed after CCs of 1 and 10 μA and also after light illumination. Both values of LRS are suddenly decreased after light turning on time of 5–10 s. The device is programmed first at 1 μA. The data retention is measured up to 1080 s. Then, light is illuminated on via-hole region for a duration of 5 or 10 s. The resistances after illumination of 5 and 10 s are approximately 1 kΩ and 100 Ω, respectively. Due to this LRS value changes with different light durations, light turned on time will control also multi-level cell operation. Unfortunately, the illumination time could not be observed directly from this data retention measurement because read out was every 60 s. However, the light turned on *time* is shown in Fig. 6a. After RESET the device by applying negative bias, the device is programmed at 10 μA. It is noticed that the LRS values are approximately 10 and 300 kΩ for the CCs of 1 and 10 μA, respectively. This is due to the smaller filament diameter at CC of 1 μA than the filament diameter at a CC of 10 μA. The data retention is measured up to 300 s. Then, suddenly, the light is turned on manually for a duration of 10 s. The LRS value changes to approximately 187 Ω. The LRS value is shown stable after light turned off up to 2000 s. After light illumination, the data retention is also continued to measure up to 2000 s. After light illumination, the LRS value does not change with time. So, the data retention is good because stronger Cu filament is formed. It shows that the LRS value changes to lower value depending on light illumination time because stronger Cu filament is formed after time being light energy stimulates Cu ion to migrate through GeSe_x_ layer. Good pulse endurance of >10^5^ times is observed after light turning on duration 10 s, as shown in Fig. 6b. Two different read voltages of 20 and −100 mV have also been applied. A read disturb is observed after 20,000 cycles at a read voltage of 20 mV and this may be due to small read voltage. The switching time could be reduced after light illumination on Ag/Ag_2_S/Au structure [15]. Figure 6c shows program/erase endurance under programming by light illumination of 10 s and erasing by RESET voltage. This P/E endurance is performed manually because of no auto-system in our lab. It is expected that longer P/E cycles can be obtained in the future. Therefore, the Cu/GeSe_x_/W CBRAM devices could be programmed by external light and erased by electrical bias on the Cu TE. Basically, the erase operation can be controlled by external current (>*I*_RESET_) and voltage (<*V*_RESET_). However, the RESET operation will have also high-speed operation of few nanoseconds [1]. So, external light impacts the device switching, which is also very useful for a new area of research.

## Conclusions

Resistive and new optical switching characteristics using thermally grown GeSe_x_ solid electrolyte in Cu/GeSe_x_/W structure have been investigated. The CBRAM device shows multi-step RESET phenomena at a CC of 300 μA with a low operation voltage of ±1.2 V, high resistance ratio of >10^4^, stable endurance of >200 cycles, and good data retention of >7 × 10^3^ s at 85 °C. Evolution of Cu filaments’ shape under CCs ranging from 1 nA to 500 μA has been understood by observing both of HRS and LRS. The device changes the HRS to LRS under white-light illumination on it, which attributes to the Cu ion migration through the GeSe_x_ solid electrolyte and form Cu metallic path. After light illumination, memory device shows good data retention of >10^3^ s and long read pulse endurance of >10^5^ cycles. This suggests that this GeSe_x_-based CBRAM device has great potential for future light-controlled optical switching and may open up a new area of research.
